# African Orphan Crops under Abiotic Stresses: Challenges and Opportunities

**DOI:** 10.1155/2018/1451894

**Published:** 2018-01-17

**Authors:** Zerihun Tadele

**Affiliations:** ^1^Institute of Plant Sciences, University of Bern, Bern, Switzerland; ^2^Center for Development and Environment (CDE), University of Bern, Bern, Switzerland; ^3^Institute of Biotechnology, Addis Ababa University, Addis Ababa, Ethiopia

## Abstract

A changing climate, a growing world population, and a reduction in arable land devoted to food production are all problems facing the world food security. The development of crops that can yield under uncertain and extreme climatic and soil growing conditions can play a key role in mitigating these problems. Major crops such as maize, rice, and wheat are responsible for a large proportion of global food production but many understudied crops (commonly known as “orphan crops”) including millets, cassava, and cowpea feed millions of people in Asia, Africa, and South America and are already adapted to the local environments in which they are grown. The application of modern genetic and genomic tools to the breeding of these crops can provide enormous opportunities for ensuring world food security but is only in its infancy. In this review, the diversity and types of understudied crops will be introduced, and the beneficial traits of these crops as well as their role in the socioeconomics of Africa will be discussed. In addition, the response of orphan crops to diverse types of abiotic stresses is investigated. A review of the current tools and their application to the breeding of enhanced orphan crops will also be described. Finally, few examples of global efforts on tackling major abiotic constraints in Africa are presented.

## 1. Introduction

Every year, natural disasters such as drought, flooding, storms, and earthquakes cause considerable damage to animals, plants, and nature in general. According to the United Nations Food and Agriculture Organization, the agricultural sector absorbs 22% of the economic impact caused by natural hazards and disasters in the developing world as agriculture contributes up to 30% of the GDP of vulnerable countries [1]. Within agriculture, the crop sector suffers the most. From 78 disasters occurring from the years 2003 to 2013, the highest damage and losses to crops were 42% followed by those to livestock (36%) [1]. According to the same report, the three most important hazards to crops are floods (60%), storms (23%), and drought (15%). The 2004 Tsunami that impacted several Asian countries, especially Thailand, and the 2011 Tsunami in Japan contributed to large losses of the agricultural land. The Japanese Tsunami alone brought high levels of salt from the sea to the agricultural land, thereby considerably affecting rice (*Oryza sativa* L.) cultivation on over twenty thousand hectares of land due to high levels of soil salinity [2].

Changes in the amount and pattern of the annual rainfall have been negatively affecting the productivity of crops in African countries especially in Ethiopia [3, 4]. The periodically occurring El Niño weather system, formed by local warming of surface water temperatures across the Central and East-Central Equatorial Pacific Ocean, is the cause of most droughts in the Horn of Africa [5, 6]. The intensity of El Niño fluctuates from year to year and is periodically severe. For instance, the El Niño in 1991/92 caused drought in about 350 million ha of land while that of 1997/98 caused drought on only 80 million ha [6]. Since the peak of El Niño development coincides with the growing season of crops in Africa [5], the effect on food security is high. As drought is usually accompanied by famine, especially in the Least Developed Countries (LDCs), the occurrence of a single natural disaster does not only affect crop and livestock production but also claims the lives of thousands or even millions of people depending on its intensity. A study in West Africa showed that the frequency of dry spells in a given year could be predicted from the annual rainfall in the previous years; hence, informed decisions can be made on the type of crop or cultivar to be cultivated and the appropriate management practices implemented [7].

Environmental stresses that cause damage to crops are broadly grouped into biotic and abiotic based on the cause of damage. While biotic stresses refer to constraints caused by living things such as pests, diseases, and weeds, abiotic stresses are those caused by climate and soil-related problems. This particular review focuses on the types and extent of damage caused by abiotic stresses and on the tools or techniques available to enhance the tolerance of indigenous crops to these environmental calamities and as a consequence to increase the productivity of crops. It gives due attention to African indigenous or orphan crops since these crops have not received much attention from the global scientific community. A changing climate, a growing world population, and a reduction in arable land devoted to food production are problems facing the world food security in the near future. The development of crops that can yield under uncertain and extreme climatic and soil growing conditions can play a key role in mitigating these problems. African indigenous crops can play a key role in this quest.

## 2. Significance of Orphan Crops in Africa

Orphan crops are also known as “neglected and underutilized species (NUS)” [60], “underutilized crops” [61], or “crops for the future” [62]. These crops are important for food security, nutrition, and income generation in many developing countries, but they have not been significantly researched [63–66]. They are often well-adapted to local growing conditions and fulfill the social and economic needs of local people and are often tolerant to many abiotic stresses compared to the world's major crops like wheat (*Triticum aestivum* L.) and maize (*Zea mays* L.) [66, 67].

### 2.1. Orphan Crops Are Extensively Cultivated in Africa

Orphan crops are staple foods for millions of people in the developing world, particularly in Africa. These crops are annually cultivated on large tracts of land and play a key role in the livelihood of the resource-poor farmers and consumers.  Table 1 shows important orphan crops, including major crops and orphan crops, in terms of the total amount of area on which they are cultivated in Africa and the total production in Africa compared to the area and production in the entire world [8]. In addition, the distribution of land and production between major and orphan crop is shown.

These crops belong to the major categories of crops such as cereals, legumes, vegetables, root crops, and fruits and have diverse centers of origin. Although similar types of crops are cultivated in the rest of the world, Africa has unique crops which are cultivated and consumed solely on this continent. Africa's unique crops include cereals such as fonio [*Digitaria exilis* (Kippist) Stapf and* D. iburua* Stapf] in western Africa, and tef [*Eragrostis tef* (Zucc.) Trotter] in the Horn of Africa, and a legume called bambara groundnut [*Vigna subterranean* (L.) Verdc.] in southern and western Africa [8].

A great deal of diversity has been maintained by farmers for diverse types of orphan or underutilized crops. Efforts have been made by national and international institutions to collect from representative locations these valuable germplasms of diverse crops. Collected germplasms are conserved at facilities in national and/or international organizations including the Svalbard Global Seed Vault in Norway where 860,00 samples of different crop species are available [68]. The Southern African Development Community (SADC) Plant Genetic Resources Center based in Lusaka, Zambia, preserved over 18,000 collections of diverse crops from 12 of its 15 member states [69]. These large germplasm collections can be used as a reliable source for screening for traits of interest. The study of only 10% of the 5000 tef collections at the Ethiopian Biodiversity Institute shows a huge diversity in several important agronomic traits which includes grain yield, harvest index, and lodging percentage [70]. Although these and other landraces possess several desirable agronomic traits, only few traits in limited crop types have so far been harnessed.

Among the root crops grown in Africa, cassava (manioc;* Manihot esculenta* Crantz), yam* (Dioscorea *spp*.)*, sweet potato [*Ipomoea batatas* (L.) Lam.], and enset [*Ensete ventricosum* (Welw.) Cheeseman] are the source of food for a large number of people. Cassava is a staple food for about 600 million people worldwide and for more than 200 million people in Sub-Saharan Africa [71]. In 2012, yam was cultivated globally on about five million hectares of which over 95% was in Africa [8]. Banana and plantain* (Musa *spp*.)* are among the major fruit crops grown in Africa. In the year 2012, about 16 million tons of banana and 27 million tons of plantain were produced in the continent [8]. Although a substantial amount of land in Africa is devoted to indigenous crops, the production constitutes a considerably low percentage of global production. For example, 10.1% of the global land devoted to barley (*Hordeum vulgare* L.) cultivation is in Africa, but the continent contributes only 4.5% of the world barley production (Table 1). This is because of the use of nonimproved cultivars as well as lack or minimal use of other agricultural inputs such as fertilizers and pesticides. The production per unit area of chickpea (*Cicer arietinum* L.), however, is slightly higher in Africa than in the rest of the world.

### 2.2. Orphan Crops Are Tolerant to Environmental Stresses

Table 2 shows some benefits of indigenous crops in terms of agronomy, nutrition, and health. Millets especially finger millet (*Eleusine coracana* Gaertn.), pearl millet [*Pennisetum glaucum* (L.) R. Br.], fonio, and tef, a tiny-seeded staple food in Ethiopia, are dominantly cultivated in semiarid areas of Asia and Africa due to their extreme tolerance to moisture deficit [72, 73]. While finger millet and pearl millet are cultivated in both continents, fonio is exclusively cultivated in West Africa. African rice* (Oryza glaberrima)*, grown in West Africa, matures early allowing it to escape terminal drought. It is also resistant to diseases and pests and tolerates fluctuations in growing conditions [45]. Tef is moderately tolerant to moisture scarcity and, in addition, it resists poorly drained soils, conditions that crops such as maize and wheat cannot withstand [74]. Noug (*Guizotia abyssinica *Cass.), an oilseed crop related to sunflower, grows best on poorly drained, heavy clay soils [75].

Legumes such as cowpea [*Vigna unguiculata* (L.) Walp.], bambara groundnut, and grass pea (*Lathyrus sativus* L.) tolerate extremely low soil moisture [10, 76]. Cowpea is the most widely cultivated food legume in Africa. In the year 2012, it was cultivated globally on 11.2 million ha of land of which 98% was in Africa, particularly in three countries (i.e., Niger; Nigeria; and Burkina Faso), which account for 82% of the total land devoted to this crop [8]. Cow pea is tolerant to drought, heat, and soils with low levels of organic matter and phosphorus [19]. Grass pea is considered as an insurance crop in Ethiopia as it produces reliable yields when all other crops fail.

Among the root crops grown in Africa, cassava, yam, sweet potato, and enset are the source of food for millions of people. Cassava is tolerant to drought and also performs better than other crops on poor soils. Enset, which is related to banana but has an underground corm as the edible part, is a major food source for over 10 million people in the densely populated regions of Ethiopia due to its extreme tolerance to drought and adaptation to diverse soil types [11].

### 2.3. Orphan Crops Provide Nutritious and Healthy Food

#### 2.3.1. Nutrition-Related Benefits

Nutritional benefits of some orphan crops are indicated in Table 2. Orphan crops often provide more nutrients than the foods that dominate global production. African cereals, particularly millets, contain high amounts of vitamins, calcium, iron, potassium, magnesium, and zinc [77]. The seeds of finger millet contain valuable amino acids especially methionine [12], which is lacking in the diets of hundreds of millions of Africans who live on starchy staples such as cassava. The seeds of fonio are nutritious especially in amino acids such as methionine and cysteine [78] which are essential for human health but deficient in major cereals such as wheat, rice, and maize [21]. Amaranth* (Amaranthus *spp*.)* which is largely consumed in Togo, Liberia, Guinea, Benin, and Sierra Leone, has a relatively high protein content and a balanced composition of essential amino acids and minerals [79].

While the grain is the main source of food for humans, crop residues, particularly the straw from cereals, are an invaluable source of livestock feed. The straw from tef is more palatable and nutritious than those of wheat and barley and, hence, it fetches higher prices [80].

Legumes such as cow pea, bambara groundnut, and grass pea are the major source of protein for consumers. Due to their ability to fix atmospheric nitrogen and convert it into a form usable for plants, legumes also contribute towards improving soil fertility. Bambara groundnut is grown for human consumption and is the third most important grain legume in Africa after cowpea and groundnut (*Arachis hypogaea* L.) [76]. The seeds of bambara groundnut are considered to be a complete food because they contain adequate quantities of protein (19%), carbohydrate (63%), and fat (6.5%) [13].

Banana, especially the orange-pulped type with high carotenoid and iron content, could reduce Iron Deficiency Anemia (IDA) by over 50% and also Vitamin A Deficiency (VAD) in East Africa, where both IDA and VAD affect a large number of people [22]. The seed oil content of noug ranges from 39.8% to 46.9% oil [75]. The fatty acid content of the oil is similar to the oils of the sunflower family with linoleic acid being the dominant oil.

#### 2.3.2. Health-Related Benefits

The benefits of some orphan crops as health-food are shown in Table 2. Finger millet is a popular food among diabetic patients because of its low glycemic index and slow digestion [27]. Finger and pearl millets were shown to have anticancer properties and might have potential in the prevention of cancer initiation [28]. This antiproliferative property is associated with the presence and content of phenolic extracts. Tef, sorghum, and millets are considered to be a healthy food since the grain does not contain gluten [25, 81, 82], the cause of celiac disease.

### 2.4. Orphan Crops Are Compatible with the Socioeconomic Conditions

Agriculture provides more than just food production—it affects nonfood products, environmental and land management, economic development, employment opportunities, social stability, and maintenance of cultural tradition and identity. Recognition of the many social and economic functions of agriculture has resulted in increased efforts of developing land use practices that support the functions in an integrated manner [83].

In southern Senegal, African rice is thought to have been domesticated 2000–3000 years ago. The Jola people of Senegal still cultivate this rice and choose the varieties to be planted based on maturation time (fast or slow maturing or both to stagger the harvest time), plant height (a tall or short variety based on harvest considerations), and soil type (the soil composition and the amount of irrigation which is particular to each field) [45]. Likewise, some fonio varieties produce grain in only 6 to 8 weeks after planting. Such varieties extend the period of time for which food is available and can bridge the gap between the last and the next harvest. Planting crops with varying maturation times increases the chances of harvesting under challenging environmental conditions [12].

In addition to adaptation to local environmental conditions, orphan or indigenous crops are preferred because of their use in preparing local recipes. For instance, tef, which is the staple food for over 60 million people in Ethiopia, is the most preferred grain since it makes the best quality spongy bread called* injera*. Compared to* injera* made from tef, those made from sorghum and maize have poor consistency and short shelf-life and thus have low acceptance by consumers. Due to these desirable properties, the grain of tef fetches higher price than that of other crops.

Similarly, indigenous crops are compatible to the agroecology of the region in which they are cultivated. The study in Northwestern Ethiopia showed that when a widely cultivated indigenous tef crop was replaced by exotic maize, the incidence of malaria increased by about 10-fold [84–86]. This was mainly due to a higher survival rate of mosquito larvae fed on maize pollen than of those fed on tef pollen.

## 3. Challenges of Abiotic Stresses to Orphan Crops

Although indigenous crops possess a variety of desirable traits, they are under continuous pressure to detect and adapt to the environmental constraints prevalent on the continent [65]. Orphan crops are normally cultivated in marginal environments in terms of climate and soil. For instance, millets are crops of dry and hot climate while tef is cultivated on poorly drained* Vertisols *in the highlands of Ethiopia where other crops fail to survive. Hence, harvests from these crops are mostly suboptimal. These constraints are broadly grouped into biotic and abiotic stresses. Biotic factors such as weeds, diseases, and insect pests cause significant crop damage in Africa due to environments conducive to these crop nuisances. The current review, however, focuses on abiotic stresses, which cause huge losses to the biomass of these crops, especially in relation to the types and extent of damage they cause and the tools developed to enhance the tolerance of crops against this damage.

Yield loss estimates have been documented for major crops. According to a website tabulating losses to these crops, yield losses due to abiotic stresses range from 50% for sugar beet to 82% for wheat [87]. However, information on orphan crops is limited.

### 3.1. Drought

A considerable amount of water is used in both rain-fed and irrigated agriculture. Irrigated agriculture accounts for about 60 percent of production in the developing countries. Although the global water withdrawal from rivers, lakes, and aquifers for agriculture is estimated at 2,000 to 2,500 km^3^ per year, only 900 km^3^ of this is used by food crops while the remaining is lost to evaporation, deep infiltration, or the growth of weeds [88]. Countries with an extensive area under irrigation utilize large amount of water. For instance, from the total water consumption in Egypt 85.9% is for irrigation, with 2.6% for industrial and the remaining 11.5% for municipal uses [89]. Since data for the majority of African countries is not available, it is difficult to know the countries which utilize small proportion of their water for agriculture. Although agriculture requires a large amount of water, the scarcity of moisture as it frequently occurs in most African countries is critical to increasing production and productivity of crops.

Semiarid and arid regions in Africa are fragile to sustain the ecosystem mainly due to the scarcity of moisture. About 40% of the population in the Sub-Saharan Africa live in arid to dry environment and the population is expected to double in the dry areas by 2050 [90]. According to these authors, crop failure in Africa is mostly not due to scarcity of precipitation but due to dry spells which occur at critical growth stages particularly during flowering.

A study on proso millet* (Panicum miliaceum)*, little millet* (Panicum sumatrense)*, foxtail millet* (Setaria italica),* and wild millet* (Setaria glauca)* showed that drought treatment before flowering contributed to a significant yield reduction in these four millets [91]. A similar study on two finger millet landraces indicated that drought occurring four weeks after sowing resulted in a complete yield loss [92].

In pearl millet, terminal drought occurring between the flowering and the crop maturity stages is the most critical as it resulted in 60% yield loss [93]. Yield losses due to drought were estimated to be 40% in tef [94], 51% in pearl millet, and 57% in bambara groundnut [95] at 50% moisture treatment.

Most orphan cereal crops in Africa such as millet and tef are C_4_ plants which efficiently utilize moisture; hence, they generally outperform C_3_ plants in hot and dry climates. A study in the Sahel region where the annual precipitation is as low as 200 mm shows that sorghum and millet had similar water use and biomass production, hence similar biomass per unit of evapotranspiration [96].

Although reports are not available for indigenous crops, the study on wheat indicated that moisture scarcity affects not only crop productivity in terms of grain yield but also the characteristics of grain including protein quality, gluten level, and dough strength [97, 98].

### 3.2. Waterlogging

Waterlogging is among the major constraints to increasing crop productivity particularly in areas which receive high precipitation and have agriculturally problematic soils. Since soil pores during waterlogging are filled with water, the diffusion of gases is hampered resulting in anaerobic conditions which might also be accompanied by the accumulation of toxic compounds. As a result, the normal functioning of stomata, photosynthesis, and roots are severely affected [99].

Waterlogging especially on* Vertisols*, the black clay soils with high water-holding capacity, restricts the cultivation of most major crops such as maize and wheat. About 43 million ha of* Vertisols* exists in 28 countries in Africa [100]. A study on millets indicated that waterlogging treatment from two weeks after sowing to crop maturity reduced the grain yield of proso millet and wild millet* (Setaria glauca)* by 16% and 18%, respectively [101].

In addition to hindering the aeration of the root systems due to poor drainage,* Vertisols* are less suitable for the cultivation of root crops such as groundnut since pulling of the pods from the soil during harvesting is hindered by the hardy nature of the soil upon drying.

### 3.3. Soil Acidity

An estimate of the total amount of land with acidic soil in the world is about 30% with about 50% of the arable land being acidic [102]. In acidic soils, aluminum toxicity is the most important factor affecting crop productivity since aluminum under a soil pH below 5 is solubilized into Al^3+^ which is toxic for plant roots, inhibiting their function and development [102]. A general rule is that aluminum concentrations between 2 and 5 ppm are toxic to aluminum-sensitive plants while concentrations above 5 ppm are toxic to tolerant species. Toxic levels of aluminum affect root growth dramatically and stunted growth, small grain size, and poor yield are the results of the consequent lack of nutrition [103]. The effects of Al-toxicity on cereal crops were recently reviewed [104].

### 3.4. Soil Salinity

Soil salinity, characterized by a high concentration of soluble salts, affects crop productivity although some indigenous crops have some level of tolerance to this abiotic stress. Soil salinity is a broad term which refers to three types of agriculturally problematic soils. These are (i) saline soil: with electrical conductivity, ECe > 4 dS/m; exchangeable sodium percentage, ESP < 15; and pH < 8.5; (ii) sodic soil: ECe < 4 dS/m, ESP > 15, and pH > 8.5; and (iii) sodic-saline soil: ECe > 4 dS/m, ESP > 15, and pH < 8.5. Chloride and sulphate salts of sodium, magnesium, and calcium are the most common soluble salts.

It is estimated that approximately 4.8% of the land in Africa is considered to be affected by salinity or sodicity [105]. Irrigated lands in the Mediterranean region have been converted into extensive salt accumulation where 60% of the farm area in Egypt is affected by saline and sodic-saline conditions [106]. Although salinity occurs due to diverse causes, the main cause of the elevated salinity level in the Mediterranean area is the use of highly saline irrigation water in the hot and dry environment where evapotranspiration is high [106].

Irrigation in arid and semiarid climates is associated with saline soils as almost all water contains some salts and, after high evapotranspiration, the salt remains on the surface of the soil and/or root zone of crops. The accumulation of salts depends on the quality of the irrigation water, the irrigation management, and the drainage of the soil. Often salinity problems are associated with poorly drained and waterlogged soils as excess water allows the salt to rise to the root zone via capillary action [107]. Once salt has accumulated, only additional water can leach the salts away from the root zone. The study in tef showed that saline soil causes up to 93% yield loss in tef [108].

### 3.5. Heat (High Temperature)

Heat causes a multitude of molecular, cellular, and physiological changes to plants. Among these, effects on respiration and photosynthesis are the major ones affecting crop productivity [109]. Extraordinary heat waves have already caused significant yield losses across the globe and pose a dire threat to global food security. Climatological extremes in general have a negative effect on plant growth and can lead to catastrophic losses in crop yields. Studies on major crops showed that, with heat, yields increase until a crop-dependent threshold (29°C for maize, 30°C for soybeans, and 32°C for cotton) but temperatures above these thresholds become very harmful [110]. Once the threshold is passed, the potential for adaptation within the crop is limited. It is estimated that a temperature increase of 3 to 4°C could cause crop yields to fall by 15–35% in Africa and Asia [109]. Modeling suggests that cereal production in Southern Africa and Southern Asia is most likely to be affected by climate change particularly by global warming [111].

### 3.6. Cold and Frost

One-third of the total global land is ice-free while 42% of land has temperatures below −20°C [112]. In these cold regions, plants have specialized adaptations that help them to survive freezing temperatures. The amount of damage caused by freezing depends on the temperature and duration of the cold stress as well as the developmental stage of the plant. Damage from cold particularly from frost is common in the highlands of the eastern Africa especially when it coincides with the growing season of crops. Although climate change is warming rather than cooling the planet, warm winters can lead to early blooming of perennials and consequent frost damage when cold conditions occur in late spring [113].

## 4. Mechanism of Crop Tolerance to Abiotic Stresses

Understanding the response of plants to resist and tolerate abiotic stresses is important for devising breeding strategies to ensure productivity under all conditions. Diverse types of mechanisms are involved in the abiotic stress tolerance of plants [114, 115]. Mechanisms employed by some indigenous crops towards adaptation and/or tolerance to diverse types of abiotic stresses are shown in Table 3. Some of these mechanisms against major abiotic stresses which affect crop productivity in Africa are briefly indicated below.

### 4.1. Drought Tolerance

Major strategies and/or mechanisms of drought tolerance or adaptation of millets that are mostly cultivated in drought-prone areas of the world were recently reviewed [72]. Four of these mechanisms are as follows: (i)* drought escape* which refers to the condition in which plants reach maturity before the drought occurs; (ii)* drought avoidance* which refers to the ability of the plant to maintain a favorable water balance under moisture stress in order to avoid water deficit in the plant tissue; (iii)* drought tolerance* which refers to the ability of the plant to produce some yield by withstanding low water potential; and (iv)* drought recovery* which refers to a condition in which plants recover from the adverse effects of drought in order to provide some yield and/or biomass.

Most African orphan crops are cultivated in a marginal environmental where moisture is limited. However, they are adapted to efficiently utilize the scarce moisture in order to sustain their growth and produce at least some yield for farmers desperately waiting for them. These neglected crops guarantee future food security either directly as alternative crops in drought-prone areas or indirectly as germplasm resources for crop improvement [116].

Several morphological and physiological traits are involved in drought tolerance of plants [117]. In little millet (*Panicum sumatrense* Roth), osmotic adjustment, which refers to the maintenance of water potential during moisture stress, and ROS (reactive oxygen species) scavenging systems are employed to protect the plant from drought [31]. Drought tolerance is known to be associated with the tensile strength of the stem of* Eragrostis* species of which tef is a member [32]. Roots also contribute to drought tolerance, and especially the efficiency of the uptake of water during reproduction and grain filling is critical since moisture scarcity during these developmental stages is detrimental to the survival of the plant [118]. The expression of dehydrin in wheat has been correlated with the acquisition of drought tolerance at early growth stage [119].

### 4.2. Waterlogging Tolerance

Plants respond to waterlogging with a variety of mechanisms [120]. In waterlogged soil, the diffusion of gases to and from the roots is severely limited. Oxygen at the root surface is severely decreased and toxic byproducts are unable to diffuse away from the plant. Many aspects of the plant response are a direct consequence of anoxia, a complete lack of oxygen or hypoxia, and reduced oxygen. One of the responses of plants to low oxygen is to change from aerobic to anaerobic metabolism as has been found for finger millet [37]. Though much less efficient than aerobic metabolism, the production of ATP by glycolysis can provide the plant with ATP for a short period of time. This requires an abundant supply of soluble sugars and thus changes in carbohydrate metabolism are often found in tolerant species. Increases in antioxidants are also observed in tolerant plants such as pigeon pea [36].

Changes in the cellular structure of the plant are also a response to waterlogging. For example, aerenchyma, air spaces in spongy tissue that let gases diffuse from the stem to the roots, is often formed in plants tolerant to waterlogging stress. These air spaces are generated either with* (lysigenous)* or without* (schizogenous)* cell death. Waterlogged sunflower developed* lysigenous* aerenchyma within two days of the application of stress [33]. Another strategy found in waterlogging tolerant species is the formation of adventitious roots when stressed [120]. Both sorghum [34] and finger millet [35] have been shown to form adventitious roots when waterlogged. A strategy to escape the flood via fast growth has been shown for mung bean [38]. In addition, plants tolerant to waterlogging possess high amount of soluble sugars [121]. In tef, the occurrence of nitrogen reductase activity in the shoots rather than in the roots has been implicated in its waterlogging tolerance.

### 4.3. Soil Acidity Tolerance

Two general mechanisms of aluminum (Al) tolerance exist in plants. These are (i)* Al exclusion*, which refers to the prevention of Al from entering the root apex, and (ii)* Al tolerance*, in which Al is allowed to enter the plant but detoxified [122]. The* Al exclusion *mechanism is also described by the exudation of organic acids or the release of phenolic compounds as well as related products which have the ability to chelate Al^3+^ ions in the rhizosphere [122].

The MATE family of proteins are transporters of metabolic and xenobiotic organic cations. Members of this family responsible for malate and citrate efflux have been implicated in aluminum resistance in wheat [123], sorghum [124], and barley [125]. An aluminum susceptible cultivar of barley was made tolerant after transformation with the TaALMT1 gene from wheat [126]. Nine barley transformant lines with the TaALMT1 gene also showed increased malate efflux and aluminum resistance and some transformants surpassed the original line in aluminum toxicity resistance [127].

### 4.4. Soil Salinity Tolerance

Three mechanisms of plant tolerance to soil salinity were reported. These are (i) tolerance to osmotic stress which reduces cell expansion in root tips and young leaves which results in stomatal closure; (ii) Na^+^ exclusion from leaf blades in order to remove the toxic effect of Na+; and (iii) tissue tolerance to accumulated Na^+^ [128].

Management of saline soils includes the provision of appropriate drainage, controlling irrigation, and the use of deep-rooted plants, which maintain the water table at a level appropriate for the land. Considerable improvements in growth and yield have been obtained by either priming seed with chemicals or extreme temperatures before planting or by applying exogenous chemicals to plants growing under salt stress. Efforts in breeding salt-tolerant cultivars are being pursued [129]. The use of saline tolerant crops both increases the amount of land available for growing and reduces the quality of the water that must be used for irrigation. Crops described as salt tolerant include date palm, asparagus, barley, and salt grass, while moderately tolerant species include olive, beet, sorghum, wheat, and rice [18].

Plant microRNAs are known to be involved in diverse abiotic stresses. A list of microRNAs from model and crop plants with differential expression under saline condition was recently documented [130].

### 4.5. Heat Tolerance

Heat stress is a major concern for crop production and much work is being done to develop plants that can still maintain yield under hot conditions. It is known that heat stress interferes with photosystem (PS) II, increases the amount of reactive oxygen species (ROS), and reduces electron transport [131]. Plants have evolved a number of adaptive, avoidance, and acclimation mechanisms to cope with heat stress.

Tolerance mechanisms involve heat shock proteins, ion transporters, osmoprotectants, antioxidants, transcription factors, and signaling molecules [132, 133]. Lipid membranes are susceptible to lipid peroxidation, and membrane thermal stability can be a selection criterion for heat stress tolerance as has been used in wheat [134]. Reactive oxygen species (ROS) are normal byproducts of cell metabolism that can be toxic at high concentrations. Many plants have systems containing antioxidants to combat ROS as protection against a wide variety of abiotic stresses.

Heat tolerant accessions have been found for pearl millet [40], chickpea [39], and cowpea [41]. A study in chickpea identified 18 SNPs from five genes (ERECTA, ASR, DREB, CAP2 promoter, and AMDH) that were significantly associated with different traits under heat and drought stress [39].

### 4.6. Cold or Frost Tolerance

Many plants increase their tolerance to cold if they are allowed to acclimate to it in low, nonfreezing temperatures. For example, rye exposed to a period of low nonfreezing temperatures can withstand temperature as low as −30°C, while the same rye without the acclimation period dies at −5°C [135]. The acclimation mechanisms include the expression of genes that function to protect membranes, the primary site of freezing injury, such as dehydrins that prevent ice nucleation [136]. In addition, CBF/DREB1 proteins, a family of transcription factors in* Arabidopsis,* control a group of genes that confer freezing tolerance [135].

Cold tolerance is the ability to grow and produce a satisfactory yield under relatively cold (but not freezing) conditions. Several sorghum accessions from Ethiopia and Uganda have been shown to be cold tolerant during both the reproductive and the vegetative stages [43]. The mechanism for this tolerance has, however, not been investigated.

Seed yield in chickpea can be increased fourfold by sowing in early winter as opposed to traditional spring sowing. However, this increases the probability of exposure to cold, necessitating cold-tolerant lines. Screening of 3276 chickpea germplasm accessions and breeding lines identified 21 cold-tolerant lines [137]. When exposed to cold stress in the reproductive phase, cold-tolerant chick peas produced viable seeds, while cold susceptible cultivars suffered from abortion due to pollen sterility [137]. The analysis of the bambara groundnut transcriptome obtained from suboptimal low temperature of 18°C showed that transcription factors such as MYB, NAC (NAM, ATAF, and CUC), and WRKY are involved in response to the stress [138].

## 5. Techniques Enhancing Abiotic Stress Tolerance in Orphan Crops

The application of modern genetic, genomic, and agronomic tools to the improvement of indigenous crops can provide enormous opportunities for ensuring global food security. A recent review indicated that there are large gaps between farmers' average yield and potential yield for several African crops [66] indicating that productivity of these crops could be boosted using improved cultivars and proper crop management practices. Recent reviews have suggested several efficient breeding and genomics techniques with immense potential in the development of crops tolerant or resistant to abiotic stresses [139–142].  Table 4 summarizes some techniques with a high potential for the improvement of African orphan crops.

### 5.1. Genetic Tools

Diverse types of genetic and breeding techniques and their applications to African orphan crop improvement were earlier reviewed [65]. Only widely implemented techniques are briefly presented below.

#### 5.1.1. Hybridization

Hybridization refers to crossing two plants of the same or related species in order to create genetic variability which is utilized to improve trait(s) of choice. Successes in hybridization resulted in semidwarf cultivars of wheat and rice, which boosted the productivity of both crops during and after the famous Green Revolution [143]. The major breakthrough in hybridization in Africa was the interspecific crossing between the Asian rice (*Oryza sativa* L.) and the African rice (*Oryza glaberrima* Steudel), which is widely cultivated in West Africa. This led to the development of the popular NERICA (New Rice for Africa) varieties. NERICA harbors desirable properties from its two parents: high grain yield and protein content from the Asian rice and tolerance to drought and poor soil fertility as well as early maturity from the African rice [45]. Due to its early maturing property, African rice escapes terminal drought, which normally occurs after the flowering stage. Hence, it is the source of food during food shortage particularly just before other crops are harvested. NERICA is currently cultivated in several African countries [144].

#### 5.1.2. Mutation Breeding

This refers to the application of either physical (such as gamma-ray and X-ray) or chemical (e.g., ethyl methane sulfonate or EMS) mutagens to alter the composition or arrangement of DNA of an organism so that mutants harboring traits of choice are selected and bred to popular cultivar(s). EMS, which mainly creates point mutations, is the most desirable mutagen as it results in a single nucleotide lesion in the gene of interest. Most mutation breeding programs aim at altering traits such as plant height and disease resistance in well-adapted plant varieties of rice, barley, and wheat. Mutation breeding is a cornerstone for releasing over 3000 globally known crop varieties including some varieties from African indigenous crops [145]. Mutation breeding and molecular breeding techniques have been successfully implemented to develop salt-tolerant crops [129].

#### 5.1.3. Plant Cell and Tissue Culture

Tissue culture techniques or* in vitro* regeneration have been widely utilized for several indigenous crops especially in tree crops due to a number of benefits. Some of these benefits are as follows: (i) a large number of planting materials can be generated from small area; (ii) planting materials are maintained free of diseases particularly from viruses; and (iii) the regeneration process is made all year round irrespective of climatic conditions since regenerates are grown in greenhouses under controlled environmental conditions. An efficient regeneration method resulting in plantlets has been developed for wild banana [146].

#### 5.1.4. Marker-Assisted Selection (MAS)

This refers to the utilization of molecular markers in breeding for traits of interest. When markers are found that are linked to genes controlling traits, the marker (not the gene) can be used to identify and select breeding stock. Markers are selected that are easily identifiable with common laboratory tests. Commonly utilized markers in crop breeding are SSRs (Simple Sequence Repeats, or microsatellites) and SNPs (Single Nucleotide Polymorphisms). SSR refers to a repeat of two to six nucleotides in the DNA sequences while SNP refers to a single nucleotide polymorphism. Other recently developed SNP-based methods include GBS (genotyping-by-sequencing) [147] and GWAS (Genome-Wide Association Studies) [148]. The potential application of GBS was recently investigated in several crops with different genome sizes and breeding systems [149]. In breeding for abiotic stress-tolerant crops, GWAS was efficiently applied in identifying aluminum resistance wheat cultivars [150].

#### 5.1.5. Transgenesis and Genome Editing

The transgenic method is the fastest adopted agricultural technology due to its high acceptance by farmers. Since the release of the first commercial transgenic crops, the annual cultivated area by transgenic crops has increased 106-fold and the production volume has increased from just 1.7 million tons in 1996 to 181 million ha in 2014 [151]. The development of cisgenesis involves a system in which plant-specific promoters are used to drive the gene of interest, instead of foreign promoters from bacteria or other organisms in case of transgenesis [152]. Genome editing especially the CRISPR/Cas9 (clustered regularly interspaced short palindromic repeats or CRISPR-associated protein 9) is an efficient and specific technique targeting multiple sites simultaneously [153, 154]. The manipulation of signaling pathways or regulatory mechanisms involved in the protection of plants against drought through this transgenic method was effective in developing plants for this particular abiotic stress [155–158].

#### 5.1.6. High-Throughput Techniques

TILLING (Targeting Induced Local Lesions IN Genomes) and EcoTILLING which screen mutagenized and natural populations, respectively, have been instrumental in identifying mutants of interest within a short period of time [159]. Both TILLING and EcoTILLING have been applied to the improvement of indigenous crops such as tef [48, 160].

### 5.2. Omics Tools

Omics techniques refer to the use of genomic, transcriptomic, proteomic, and related techniques for improving crops. Omics tools provide useful information regarding the response and adaptation of plants to abiotic stresses [161, 162]. Genomic tools have proven to be robust in developing crop varieties with improved traits [163]. A list of genes known to be involved in diverse abiotic stresses and crops has been documented for various crops [164, 165]. The genome sequencing of an orphan crop tef has been utilized in the investigation of drought and nutrition-related genes [166].

Some genes are known to be involved in multiple abiotic stresses tolerance. Among these, NAC transcription factor enhances both drought and salt tolerance [167, 168], while RCI2/PMP3 (which encodes small membrane proteins of the PMP3 family) and bZIP (Basic leucine zipper) transcription regulate several abiotic stresses [169, 170].

RNASeq, quantitatively sequencing mRNA using next-generation sequencing technologies, has in recent years become a standard; for example, gene expression profiling of chickpea responses to drought, cold, and high-salinity has been performed [171, 172].

MicroRNAs, a class of small noncoding RNAs that are between 21 and 24 nucleotides long, are associated with responses to diverse abiotic stresses including drought, salinity, extreme temperatures, nutrient deprivation, and heavy metals [173]. miR169 is one of the largest miRNA families that is conserved in all plant species and significantly contributes to proper plant development and in plant response to environmental stress [174]. The study in foxtail millet* (Setaria italica)* showed that, after exposing the plant to moisture scarcity, 32 dehydration-responsive miRNAs were upregulated in tolerant cultivar and 22 miRNAs were downregulated in sensitive cultivar, suggesting that miRNA-mediated molecular regulation might play important roles in providing contrasting characteristics to these cultivars [175]. Successes in developing abiotic stress tolerance in crop plants using either the overexpression or downregulation of the miRNAs and their targets were reviewed [173, 174]. Although reports for African indigenous crops are not yet available, microRNAs induced in drought have been reported for rice [176].

Proteomic tools have also been implemented to study drought tolerance in rice [177] and salinity and drought tolerance in cereals [178].

### 5.3. Agronomic Tools

Throughout the world, roughly 1.5 billion people live in risk-prone, marginal environments and are not using modern agricultural technologies. New management systems that can adapt to local, highly variable small farms typical of the developing world are necessary [179]. Agroecology, the study of the ecology of agricultural systems, has brought novel land management approaches to subsistence farmers. Several agronomic techniques have been developed which enhance the tolerance or adaptation of crops to diverse types of abiotic stresses. Four of these techniques are briefly discussed below.

#### 5.3.1. Conservation Tillage

Conservation tillage, also known as minimum tillage or no-till, is widely used in some parts of the world mainly to control erosion and increase soil fertility. Conservation tillage, leaving the previous year's crop residue on the fields, has the effect of reducing soil erosion and runoff. A study in drought-prone areas of northern Ethiopia showed that conservation tillage enhanced the availability of soil moisture to sorghum and chickpea [50]. Similarly, in Zimbabwe's semiarid areas receiving low amounts of rainfall, conservation tillage increased the productivity of maize compared to the conventional tillage [180].

#### 5.3.2. Proper Drainage of Waterlogged Soils

As indicated above, waterlogging is among the major constraints to crop production in some parts of Africa. The broad-bed maker (BBM), a low-cost modification of the traditional Ethiopian plow, has been efficiently utilized to drain excess moisture from fields containing* Vertisols* through the creation of broad-beds and furrows (BBF). Due to the improved drainage obtained using BBM, a grain yield increase of 78% and straw yield increase of 56% were obtained for wheat in the central highlands of Ethiopia [53].

#### 5.3.3. Presowing Seed Treatments to Boost Tolerance to Salts

Since seed germination and the early establishment of seedlings are the stages of plant development most sensitive to salt stress, rapid and uniform germination is required [129]. Enhanced growth can be obtained by immersing seeds in salt solution before sowing. This process is commonly known as “priming.” Five types of priming are known: (i)* osmopriming* in which seeds are treated with solutions of sugars, glycerol, or mannitol that promote germination; (ii)* halopriming *involving soaking of seeds in solutions of inorganic salts such as MnSO_4_; (iii)* hydropriming* in which seeds are soaked in water before sowing; (iv)* thermopriming* involving treating seeds with high or low temperatures before sowing; and (v)* hormone priming*, which involves the use of plant growth regulators such as auxin, gibberellic acid, and ethylene.

#### 5.3.4. The System of Crop Intensification (SCI)

SCI was formed in order to transfer the knowledge and experience developed by System Rice Intensification (SRI) to diverse crops. SRI has been tested in 50 countries and coordinated by the International Programs of Cornell's College of Agriculture and Life Sciences. SCI involves improving planting and growing techniques such that more yield can be obtained using fewer seeds and less water through management of the relationship between the plant and soil. Plant spacing and age of rice at transplantation time are two examples of this kind of management. An application of this technique to abiotic stresses is the reduction of toxic levels of inorganic arsenic in rice fields by using the optimum amount of water instead of excess water [181, 182]. In addition to major crops such as rice and wheat, SCI gave positive results for indigenous crops such as finger millet, tef, and mustard [54].

### 5.4. Plant Growth Regulators or Hormones

Several plant growth regulators (PGRs) play a key role in the plant response to diverse environmental conditions especially to abiotic stresses. Major PGRs including abscisic acid (ABA), cytokinin, ethylene, jasmonate (JA), auxin, brassinosteroids (BRs), and gibberellins (GAs) are known to be involved in the protection of plants against salt stress [55]. Exogenous application of Methyl jasmonate (MeJA) protects plants from both biotic and abiotic stresses [183]. Jasmonates are also involved in the protection of plants from both heat and cold [56]. Protection from the cold is through the regulation of C-repeat binding factor (CBF) by jasmonate during cold stress [56]. A recent study on orphan cereals showed that deficiency in gibberellic acid did not only reduce the height of tef and finger millet but also enhanced the tolerance of these crops to drought [184]. A study on a model plant* Arabidopsis* showed that strigolactones, a small class of carotenoid-derived compounds, play a positive regulatory role in the plant response to drought and salt stress [185].

## 6. Initiatives to Mitigate Abiotic Stresses in Africa

A number of projects have been initiated to improve productivity of crops through tackling major constraints. Among these, three initiatives relevant to Africa are briefly discussed below.

### 6.1. Stress-Tolerant Rice for Africa and South Asia (STRASA)

STRASA was formed in 2007 by IRRI (International Rice Research Institute) and Africa Rice to develop and deliver rice varieties tolerant to diverse abiotic stresses in the unfavorable rice-growing environments [57] (Table 5). The initiative focused on developing rice varieties tolerant to drought, flooding (submergence), soil salinity, iron toxicity, and cold. Improved rice varieties tolerant to drought, flooding, and salinity have been extensively investigated in South Asian countries such as Bangladesh, India, and Nepal while those tolerant to iron toxicity have been tested in several African countries where a single variety has been released in Sierra Leone. The cold-tolerant lines have been evaluated in cold-prone African countries where a total of seven improved varieties have been released in Burundi, Madagascar, and Mali.

### 6.2. Drought-Tolerant Maize for Africa (DTMA)

DTMA was formed by CIMMYT (International Maize and Wheat Improvement Center) and IITA (International Institute of Tropical Agriculture) in close collaboration with private and public sectors where they developed over 200 maize varieties for 13 African countries, namely, Angola, Benin, Ethiopia, Ghana, Kenya, Malawi, Mali, Mozambique, Nigeria, Tanzania, Uganda, Zambia, and Zimbabwe (Table 5). In these regions where maize significantly suffers from moisture scarcity, about 54,000 metric tons of certified drought-tolerant seed was produced through the program in a single year [58, 186]. Countries which benefited a lot from the program by releasing maximum number of varieties are Nigeria (21), Zambia (18), and Malawi (17).

### 6.3. Feed the Future Innovation Lab

Feed the Future, the US Government's global hunger and food security initiative, addresses the global agricultural challenges through its 24 Feed the Future Innovation Labs [59] (Table 5). Five programs related to developing climate-resilient crops and focus countries are on beans (Haiti, Honduras, Malawi, Mozambique, Tanzania and Zambia), on chickpea (Ethiopia, India and Turkey), on cowpea (Burkina Faso, Ghana, Nigeria and Senegal), on millet (India, Mali and Nigeria) and on sorghum (Ethiopia, India, Mali and South Africa). From the above 15 countries in the resilient program, about 75% are from Africa from which three countries (Ethiopia, Mali and Nigeria) are represented each in two programs. In general, this shows a high level of commitment by scientists and government of USA to contribute to food security in Africa through tackling major climatic and soil constraints.

## 7. Conclusions and Prospects

A variety of abiotic stresses cause considerable crop yield loss annually. Compared to major or exotic crops, orphan crops are more tolerant to locally prevailing abiotic stresses. However, even these resilient orphan crops do not produce optimum yields when subjected to abiotic stresses. Although indigenous crops are better protected than major crops of the world against certain abiotic stresses, the effects on orphan crops are substantial, especially under extreme moisture scarcity. African researchers are faced with the challenge of improving the productivity of orphan crops through tackling major yield limiting factors, which include a variety of abiotic stresses. Moreover, researchers and policy makers need to prioritize research directions based on the urgency and extent of the prevailing abiotic stresses. Useful lessons could also be learned from the work made in other parts of the world. For instance, in Japan, a salt-tolerant rice variety called “kaijin” was developed in a very short period through mutation breeding and TILLING in response to the large areas of land devoted to rice cultivation that were affected by the high level of salts from the sea during the 2011 Tsunami [2]. Global warming and climate change call for the development of climate-smart crops. Definition of the optimum ideotype for each crop type is necessary so that breeders focus on developing crop varieties with a desirable architecture and high yield potential. Both conventional and modern breeding techniques as well as omics tools play key roles in tackling the problems of abiotic stresses. Once candidate lines with desirable abiotic stress tolerance have been obtained, increased tolerance to other abiotic stresses or enhanced productivity can then be obtained through hybridization. Some key achievements from the global initiatives are contributing to increased productivity of crops cultivated in Africa. However, further commitments are required to include major staple crops in the continent which are largely neglected from global scientific community.

## Figures and Tables

**Table 1:** Major crops grown in Africa with their global area of cultivation, total production, and productivity. *∗* indicates the top 10 globally important crops in terms of total production. *∗∗* refers to finger millet, pearl millet, and proso millet and others. Source: [8, 9].

Crop		Center of origin	Area cultivated in Africa		Total production in Africa		Yield (t ha^−1^)	
Common name	Botanical name		(000′ ha)	% of global	(000′ tons)	% of global	Global	Africa
*Cereals*								
Barley^*∗*^	*Hordeum vulgare*	Ethiopia	4,997	10.1	6,022	4.5	2.7	1.2
Fonio	*Digitaria exilis*		566	100	587	100	1.0	1.0
Maize^*∗*^	*Zea mays*	Mexico & Central America	33,710	19.0	69,636	7.9	4.9	2.1
Millets^*∗∗*^	Diverse species	Ethiopia	19,977	62.9	16,083	53.8	0.9	0.8
Rice^*∗*^	*Oryza sativa*	India	10,538	6.4	26,823	3.7	4.4	2.5
Sorghum	*Sorghum bicolor*	Ethiopia	23,169	60.7	23,312	40.9	1.5	1.0
Tef	*Eragrostis tef*	Ethiopia	3,000	100	4,700	100	1.6	1.6
Wheat^*∗*^	*Triticum *spp.	Middle East	10,216	4.7	24,710	3.7	3.1	2.4
*Food legumes (pulses)*								
Bambara beans	*Vigna subterranea*		232	100	159	100	0.7	0.7
Beans, dry	Diverse species	Diverse	7,829	26.7	5,069	21.5	0.8	0.6
Chickpea	*Cicer arietinum*	Central Asia	593	4.8	696	5.9	0.9	1.2
Cowpea	*Vigna unguiculata*	India	10,402	92.1	5,402	94.5	0.5	0.5
Pigeon pea	*Cajanus cajan*	India	754	14.1	632	14.9	0.8	0.8
*Vegetables*								
Cabbages	*Brassica *spp.	China	124	5.2	2,805	4.0	29.3	22.6
Okra	*Abelmoschus esculentus*	Ethiopia	516	47.4	1,833	21.9	7.7	3.5
Tomato^*∗*^	*Lycopersicon lycopersicum*	Mexico & Central America	1,010	21.0	17,937	11.1	33.7	17.8
*Oil seeds*								
Castor seed	*Ricinus communis*	Ethiopia	250	14.8	90	4.6	1.1	0.3
Sesame	*Sesamum indicum*	Central Asia	3,283	41.5	1,708	42.3	0.5	0.5
Soybean^*∗*^	*Glycine max*	China	1,637	1.5	1,986	0.8	2.3	1.2
Sunflower	*Helianthus annuus*	North America	1,790	7.2	2,223	5.9	1.5	1.2
*Roots & tubers*								
Cassava^*∗*^	*Manihot esculentum*		13,662	67.0	149,479	52	12.9	10.9
Enset	*Ensete ventricosum*		312	100	728	100	2.3	2.3
Potato^*∗*^	*Solanum tuberosum*	North America	1,894	9.9	28,169	7.7	18.9	14.8
Sweet potato^*∗*^	*Ipomoea batatas*	Mexico & Central America	3,506	43.3	17,999	17.4	12.7	5.1
Taro	*Colocasia esculenta*	India	1,127	85.7	7,367	73.8	7.6	6.5
Yam	*Dioscorea *spp.	India	4,801	95.3	56,500	96.1	11.7	11.7
*Fruits*								
Banana^*∗*^	*Musa *spp.	India	1,493	30.1	15,863	15.5	20.6	10.6
Mango	*Mangifera indica*	India	691	13.4	7,135	16.9	8.1	10.3
Papaya	*Carica papaya*	Mexico & Central America	134	30.8	1,330	10.7	28.5	9.8
Plantain	*Musa *spp.		4,368	80.8	26,545	71.4	6.9	6.1
*Others*								
Cocoa	*Theobroma cacao*	Mexico & Central America	6,337	63.8	3,289	65.7	0.5	0.5
Coffee	*Coffea *spp.	Ethiopia	1,990	19.8	1,000	11.3	0.9	0.5

**Table 2:** Diverse types of benefits related to orphan African crops.

Beneficial trait	Crops	Reference
*Agronomy-related*		
Drought-tolerant	African rice, bambara groundnut, cassava, cowpea, enset, Ethiopian mustard, grass pea, pearl millet, tef, yam	[10–16]
Waterlogging tolerant	Tef	[17]
Salinity-tolerant	Sorghum (moderately tolerant)	[18]
Heat-tolerant	Cow pea, pearl millet	[19]
Adaptation to poor soil	African rice, cassava, cowpea	[12, 14, 19]
Early maturing (drought escape)	African rice, amaranth, fonio	[12, 13]
Erosion control (fast ground cover)	Cowpea	[20]
Disease tolerance	African rice, Ethiopian mustard, tef	[12, 16, 17]
Pest tolerance	African rice, Ethiopian mustard, tef	[12, 16, 17]
*Nutrition-related*		
Complete food (protein, carbohydrate, fate)	bambara groundnut	[13]
Rich in essential amino acids	Amaranth, bambara groundnut, cowpea, finger millet, fonio, grass pea	[10, 12, 13, 21]
Rich in iron	Banana, fonio, tef	[12, 22]
High vitamin A content	Banana	[23]
High oil content	Noug [*Guizotia abyssinica* (L.f.) Cass.]	[24]
*Health-related*		
Gluten-free	Tef, millets	[25, 26]
Low glycemic index	Finger millet	[27]
Anticancer property	Millets	[28]

**Table 3:** Mechanisms of orphan crops tolerance to diverse abiotic stresses.

Abiotic stress	Trait	Response to stress	Crop	Reference
Drought	Seed yield	High yield for drought-tolerant genotypes	Pearl millet	[29]
	Flowering time	Adjust phenology to rainfall pattern	Pearl millet	[30]
	Shoot length	Decreased under drought	Little millet	[31]
	Root length	Increased under drought	Little millet	[31]
	Leaf tensile strength	Increased in drought-tolerant plants	Tef	[32]
	Water extraction	Less extraction before flowering; more extraction after flowering	Pearl millet	[29]
	Chlorophyll content	Decreased under drought	Little millet	[31]

Waterlogging	Cellular structure	Aerenchyma formation	Sunflower	[33]
		Formation of adventitious roots	Sorghum, finger millet	[34, 35]
	Metabolism	Generation of antioxidants	Pigeon pea	[36]
		Change to anaerobic metabolism	Finger millet	[37]
	Plant height	Fast growth	Mung bean	[38]

Heat	Growth	Growth and nutrient uptake	Chickpea, Pearl millet	[39, 40]
	Yield	Unknown	Cowpea	[41]

Cold and frost	Growth	Early flowering, protracted rosette	Faba bean	[42]
		Early flowering	Sorghum	[43]

**Table 4:** Major techniques applied to develop abiotic stress-tolerant African orphan crops.

Technique	Improved trait	Crop	Reference
*Breeding & genomics*			
Selection/screening	Soil acidity	Tef	[44]
Hybridization (intra- & interspecific)	Drought tolerance	NERICA rice	[45]
Mutation breeding	Diverse traits	Tef	[46]
Marker-assisted method	Drought tolerance	Pearl millet	[47]
TILLING (Targeting Induced Local Lesions IN Genomes)	Several traits	Chickpea, millets, tef	[48, 49]
*Agronomy & physiology*			
Conservation tillage	Drought tolerance	Sorghum and chickpea	[50]
Biochar application	Soil acidity tolerance; improved soil properties	Tef & other crops	[51, 52]
BBF (broad-beds & furrows)	Improved drainage	Faba bean and tef	[53]
SCI (System of Crop Intensification)	Several	Finger millet, tef, mustard	[54]
Plant hormones	Salt & cold tolerance	Several crops	[55, 56]

**Table 5:** Selected projects focused on tackling diverse types of abiotic stresses in Africa. STRASA: Stress-Tolerant Rice for Africa and South Asia; DTMA: Drought-Tolerant Maize for Africa; FARMD: Forum for Agricultural Risk Management in Development.

Name	Crops	Abiotic stress targeted	African country of focus	Achievement/improved varieties	Reference
STRASA	Rice	Drought	Burundi, Guinea Conakry, Madagascar, Mali, Nigeria & Sierra Leone	20 varieties	[57]
		Submergence	Mali	2 varieties	
		Salinity	Gambia & Sierra Leone	6 varieties	
		Cold	Burundi, Madagascar & Mali	7 varieties	
		Iron toxicity	Sierra Leone	1 variety	

DTMA	Maize	Drought	Angola, Benin, Ethiopia, Ghana, Kenya, Malawi, Mali, Mozambique, Nigeria, Tanzania, Uganda, Zambia & Zimbabwe	Improved drought-tolerant maize varieties	[58]

Feed the Future Innovation Labs	Beans	Drought & heat	Malawi, Mozambique, Tanzania & Zambia		[59]
	Chickpea		Ethiopia		
	Cowpea	Drought	Burkina Faso, Ghana, Nigeria & Senegal		
	Millet	Drought & heat	Mali & Nigeria		
	Sorghum	Drought & heat	Ethiopia, Kenya, Mali & South Africa		
